# Mapping pathogenic bacteria resistance against common antibiotics and their potential susceptibility to methylated white kidney bean protein

**DOI:** 10.1186/s12866-024-03202-x

**Published:** 2024-02-05

**Authors:** Mahmoud Sitohy, Gamal Enan, Seham Abdel-Shafi, Neveen Abou El-Wafa, Nashwa El-Gazzar, Ali Osman, Basel Sitohy

**Affiliations:** 1 Biochemistry Department, Faculty of Agriculture, Zagazig University, Zagazig, 44511 Egypt; 2 Botany and Microbiology Department, Faculty of Science, Zagazig University, Zagazig, 44519 Egypt; 3 Department of Clinical Microbiology, Infection and Immunology, Umeå University, 90185 Umeå, Sweden; 4 Institution of Diagnostic and Intervention, Oncology, Umeå University, SE-90185 Umeå, Sweden, Umeå University, Umeå, 90185 Sweden

**Keywords:** Methylated protein, Antibiotic effectiveness, AST, MDR, FTIR

## Abstract

As antibiotics cannot inhibit multidrug-resistant bacteria (MDR), continuous research is mandatory to find other antibacterials from natural resources. Native legume proteins and their modified forms exhibited broad spectra of high antimicrobial activities. Sixteen bacterial isolates were mapped for antibiotic resistance, showing resistance in the range of (58–92%) and (42–92%) in the case of the Gram-negative and Gram-positive bacteria, respectively. White native *Phaseolus vulgaris* protein (NPP) was isolated from the seeds and methylated (MPP). The MIC range of MPP against 7 MDR bacteria was 10–25 times lower than NPP and could (1 MIC) considerably inhibit their 24 h liquid growth. MPP showed higher antibacterial effectiveness than Gentamycin, the most effective antibiotic against Gram-positive bacteria and the second most effective against Gram-negative bacteria. However, MPP recorded MICs against the seven studied MDR bacteria in the 1–20 µg/mL range, the same for Gentamycin. The combination of Gentamycin and MPP produced synergistic effects against the seven bacteria studied, as confirmed by the Transmission Electron Microscopic images. The antimicrobial activity of MPP against the seven MDR bacteria remained stable after two years of cold storage at 8–10 °C as contrasted to Gentamycin, which lost 20–72% of its antimicrobial effectiveness.

**Supplementary Information:**

The online version contains supplementary material available at 10.1186/s12866-024-03202-x.

## Background

Multidrug-resistant bacterial MDR is an evolutionary response of bacteria to withstand commonly used antimicrobial drugs. Currently, they are generally frequently impairing probably the management of worldwide infectious diseases and thus triggering a significant concern in public health since they can avoid the action of antibiotics subjecting many illness cases to mortality risk. However, there is still no description of these MDR bacteria or known procedure to combat them. This work is an initial trial to specify and delineate some of these bacteria within the current environmental context as a first step. The second step is to test the potential susceptibility of these bacteria to some natural antibacterial product, i.e., methylated white kidney bean (MPP). The study revealed that currently used antibiotics lost most antibacterial activity, except for a few examples, including Gentamycin, which is, the most effective. MPP showed higher antibacterial effectiveness than Gentamycin. The combination between Gentamycin and MPP produced synergistic effects against the seven studied bacteria. The antimicrobial activity of MPP against the seven MDR bacteria remained stable after two years of cold storage at 8-10 °C as contrasted to Gentamycin, which lost 20-72% of its antimicrobial effectiveness.

## Introduction

Antimicrobial-resistant bacteria trigger a significant concern in public health [1]. Antimicrobial and multidrug-resistant bacterial (MDR) strains are frequent in hospitals, probably impairing the worldwide infectious disease management [2]. MDR is an evolutionary response of bacteria to withstand commonly used antimicrobial drugs [3]. It is one of the most crucial issues associated with mortality and economic loss [4]. Increased mortalities are currently attributed to nosocomial infections with antimicrobial resistance, presaging dire consequences in the future [5]. Approximately 0.7 million deaths occur yearly from MDR [6], accentuating the need to search for other antimicrobials based on safe, natural agents such as plant extracts, nano-particles and modified legume proteins for single or antibiotic-combined use [7, 8]. MDR bacteria resisted various antibiotics due to their efflux pumps, cell wall structure, or porins. The drug resistance mechanisms include severe mutations in existing genes or the acquisition of emergent antibiotic-resistance genes through horizontal gene transfer, which is highly responsible for resistant microbial pandemics [9]. Nevertheless, antimicrobial resistance is believed to have existed before the invention of antibiotics and its unlimited application in animal husbandry, hospitals, and low-income countries [10]. Colistin-resistance genes were reported in *Pseudomonas aeruginosa, Acinetobacter baumannii,* and Enterobacteriaceae, while methicillin resistance gene was detected in *Staphylococcus aureus* and vancomycin resistance gene in *Staphylococcus aureus* and *Enterococci* [11] Plasmid-mediated genes are responsible for the spread of carbapenemases in different bacteria [12]. Thus, the deteriorating effectiveness of antibiotics is becoming a severe challenge for modern medicine.

Natural plant extracts provide diversified sources of chemical compounds with variable therapeutic applications, comprising antiviral, antibacterial, antifungal, and anticancer activities [13]. Basic proteins, spices, herbs, and herbal extracts have been reported for their antimicrobial activities for several years [14]. Antimicrobial peptides and proteins (AMPPs) are distinct lead agents counteracting microbial resistance. Although some antimicrobial peptides may still be far from the standard potency of current antibiotics, they remain highly promising based on their ability to avoid developing bacteria resistance mechanisms and their relatively low toxicity. Generally, AMPPs contain high levels of cationic amino acids [15, 16], enabling nonspecific binding to biological membranes [17, 18]. In 2012 [19] reported, considerable antibacterial activities of soybean seed glycinin and basic subunit equivalent to or higher than penicillin.

Nevertheless, the total leguminous seed protein was either void of or showing very low antibacterial activities. So, research efforts are intended to enhance or create this activity either through releasing bioactive peptides by enzymatic hydrolysis [20] or through chemical modification to augment the protein-positive charges via esterification. Esterification is a well-known technique that can enhance the net positive charges on the surface of the modified proteins, imparting them with antibacterial properties [21]. This chemical modification proved effective in enhancing the antibacterial activities of native proteins [22]. In the current study, the potential antibacterial activity of methylated white kidney bean (*Phaseolus vulgaris*) protein was tested against the most multidrug-resistant bacteria after being mapped using the antibiotic sensitivity disc method.

## Material and Methods

### Bacterial strains and media

The data in Table 1 lists the pathogenic bacteria used in this study. These bacteria were maintained as frozen stocks at -20C° in glass beads and were prepared in Brain Heart infusion (BHI) broth (Oxoid). The listed bacteria were used in the antibiotics susceptibility test (AST). Out of this collection, 2 Gram-positive bacteria viz *Streptococcus pyogenes* LMG21599 (*S. pyogenes*) and *Staphylococcus pasteuri* [23] (*S. pasteuri*) and 5 Gram-negative viz *Salmonella* enterica subsp. enterica, serovar Typhimurium strain LMG10395 (*S.*Typhimurium), *Klebsiella pneumonia* (*K. pneumonia*) [24], *Klebsiella oxytoca* LMG3055 (*K. oxytoca*), *Escherichia coli* LMG8223 (*E. coli),* and *Pseudomonas aeruginosa* LMG8029 (*P. aeruginosa*) showed the best responses to the tested protein preparations. All the bacteria used were provided from the culture collection of Botany and Microbiology Department, Faculty of Science, Zagazig University, Egypt. After propagation into BHI broth (Oxoid), these bacteria were kept on BHI agar slopes at 4 °C until subculturing on the same media,i.e., BHI agar (Oxoid) every month.

**Table 1:** Bacterial strains used as indicators and their sources

Tested bacteria	Source	Additional information
Gram-positive		
*Staphylococcus aureus*	DSM1104	Deutsche Sammlung von Mikroorganismen und Zellkulturen, GmbH, Braunschweig, Germany
*Staphylococcus pasteuri*	(OSC)^a^	(Mahmoud et al., 2023) [23]
*Streptococcus pyogenes*	LMG21599	laboratorium voor mikcrobiologie, Gent culture collection, Universiteit Gent, Belgium
*Staphylococcus aureus*	(OSC)	
*Bacillus cereus*	ATCC14579	American Type Culture Collection, Rock ville, Maryland, USA
Gram-negative		
*Klebsiella pneumonia*^1^	(OSC)	(Askoura et al., 2021) [24]
*Klebsiella pneumonia*^2^	(OSC)	(Askoura et al., 2021) [24]
*Klebsiella pneumonia*^*3*^	(OSC)	(Askoura et al., 2021) [24]
*E. coli*^*1*^ (uR10)	(OSC)	
*E. coli*^2^	LMG8223	laboratorium voor mikcrobiologie, Gent culture collection, Universiteit Gent, Belgium
*E. coli*^3^ (uR4)	(OSC)	
*K. oxytoca*	LMG3055	laboratorium voor mikcrobiologie, Gent culture collection, Universiteit Gent, Belgium
*S*. Typhimurium	LMG10395	laboratorium voor mikcrobiologie, Gent culture collection, Universiteit Gent, Belgium
*P. aeruginosa*	LMG8029	laboratorium voor mikcrobiologie, Gent culture collection, Universiteit Gent, Belgium
*Shigella sp*	(OSC)	It was isolated and identified in their study from 8 years old male stool
*P. mirabilis*	DSM4479	Deutsche Sammlung von Mikroorganismen und Zellkulturen, GmbH, Braunschweig, Germany

^a^(OSC): Our Strain Collection. Three isolates from *Klebsiella pneumonia* and *E. coli* were denoted by the superscript numbers ^1,2^ and ^3^

## Plant Material

White Kidney bean (*Phaseolus vulgaris*) seeds were purchased from the local market (Zagazig, Sharkia Governorate, Egypt).

## Isolation, esterification, and chemical characterization of *Phaseolus vulgaris* protein

### Sample preparation

White kidney bean seeds were manually separated from impurities and milled into powder using a Moulinex mixer (Type 716, France) set to its highest speed to pass through a 1 mm2 sieve. The powder was then continuously defatted with n-hexane for 8 h. A rotary evaporator was used to evaporate the solvent, and the dry, defatted meal was kept at 4 °C.

### Protein isolate extraction

Protein isolate was recovered from *Phaseolus vulgaris* seeds using Fan and Sosulski's procedure [25] with a few changes. NaOH (0.1 N) was used to bring 5% (w/v) defatted *Phaseolus vulgaris* flour suspensions in water to a pH 9, after which they were agitated for an hour and centrifuged for 15 min at 2000 xg. The extracts were collected, and the pH was lowered to 4.5 with (1 N) HCl to precipitate the proteins. The proteins were separated by centrifuging at 2000 xg for 15 min, and removing the supernatant. The residue was washed in a distilled water system, then dispersed in a limited volume of water at pH 7.5, dialyzed for 48 h, and lyophilized.

### Esterification of native protein

Methylated *Phaseolus vulgaris* protein (MPP) was prepared from native *Phaseolus vulgaris* protein (NPP) via the esterification method following [21, 26]. The esterification of proteins was measured as described by [27].

### Chemical characterization

The protein pH-solubility curves of MPP and NPP were measured following [28]. As described previously [8], urea-PAGE and SDS-PAGE of MPP and NPP were carried out. An FT-IR spectrometer (Nicolet Nexus 470, DTGS, Thermo Scientific, Waltham, MS, USA) was used to estimate the infrared spectra of (MPP, NPP) at 25 °C using the potassium bromide (KBr) pellet method [29].

## Antibiotic susceptibility test (AST)

The procedures adopted by Reller [30] were followed. An inoculum of the indicator bacteria was prepared (2 × 10^5^ CFU mL^−1^ for each strain), placed by sterile automatic pipette (Promega, USA) and spread onto Muller-Hinton agar plates. Thus, 12 antibiotics described in (Table 2 and 3) were put onto the surface of the Mueller–Hinton agar. The plates were incubated at 35 °C for 24-48 h. Inhibition zones were measured using a millimeter ruler and results were recorded according to the Laboratory Standards Institute (CLSI). Sensitivity or resistance was expressed as Balouiri [31] based on the guidelines of CLSI M100 [32].

**Table 2:** Gram-positive bacterial resistance and antibiotic effectiveness against the tested bacteria

Antibiotics	Category &inhibition zone (mm)^a^		Classification of Tested bacteria (**S, I & R**)^b^ according to Inhibition zone (mm)					% Antibiotic effectiveness (**AE**)^c^
	S	*R*	***Staph pasteuri***	***Staph aureus***	***Bacillus cereus***	***Staph aureus (OSC)***	***S. pyogenes***	
**Ciprofloxacin** (5µg)	(≥ 21)	(≤ 15)	S (22)	S (26)	S (26)	R (9)	R (0)	60%
**Chloramphenicol**(30µg)	≥ 18	≤ 12	S (20)	S (24)	I (14)	R (11)	S (22)	60%
**Vancomycin**(30µg)	≥ 12	≤ 16	S (12)	R (7)	R (8)	R (1)	R (2)	20%
**Gentamicin** (10 µg)	≥ 15	≤ 12	S (20)	S (22)	I (14)	S (16)	S (17)	80%
**Oxacillin** (1µg)	≥ 22	≤ 21	R (16)	R (8)	R (0)	R (0)	R(2)	0%
**Azithromycin**(15µg)	≥ 18	≤ 13	S (20)	R (0)	R (6)	R (0)	I (15)	20%
**Rifampin** (5µg)	≥ 20	≤ 16	R (8)	S (26)	R (2)	R (0)	R (4)	20%
**Nitrofurantoin** (300µg)	≥ 17	≤ 14	R (10)	S (18)	R (7)	R (0)	R (14)	20%
**Trimethoprim** (5µg)	≥ 16	≤ 10	S (18)	R (10)	R (0)	R (0)	R (0)	20%
**Tetracycline** (30 µg)	≥ 19	≤ 14	S (18)	S (17)	R (12)	R (0)	R (10)	40%
**Clindamycin** (2µg)	≥ 21	≤ 14	R (10)	R (4)	R (8)	R (0)	R (0)	0%
**Linezolid** (30µg)	≥ 21	≤ 20	R (12)	S (26)	R (16)	R (10)	R (6)	20%
**MAR INDEX**^d^			**0.42**	**0.42**	**0.75**	**0.92**	**0.75**	

^a^(EM100 connect – CLSI M100 ED30, 2020)

^b^*S, I & R* (Sensitive, Intermediate & Resistant)

^c^*AE* Antibiotic effectiveness % = (No of Sensitive strain/total No of strain)

^d^Multi antibiotics resistant MAR = a/b, where a represents the number of antibiotics to which the test isolate depicted resistance and b represents the total number of antibiotics to which the test isolate has been evaluated for susceptibility

**Table 3:** Gram-negative bacterial resistance and antibiotic effectiveness of Gram-negative specific antibiotics against the tested bacteria

Antibiotics disk	Category &inhibition zone (mm)^a^		Classification of Tested bacteria (**S, I & R**)^b^according to Inhibition zone (mm)											% Antibiotic effectiveness (**AE**)^c^
	**S**	**R**	***E. c1***	***E. c2***	***E. c3***	***K. p1***	***K. p2***	***K. p3***	***K. oxytoca***	***S.* Typhimuriam**	***Shigella sp***	***P. aeruginosa***	***Pro mirabilis***	
**Ampicillin – sulbactam**(20 µg)	≥ 15	≤ 13	R (0)	R (0)	R (0)	R (0)	R (0)	R (0)	R (0)	R (2)	R (2)	R (0)	R (1)	0%
**Cefepime** (30 µg)	≥ 25	≤ 18	R (0)	R (0)	R (0)	R (0)	R (0)	R (0)	R (0)	R (4)	R (3)	R (0)	R (1)	0%
**Meropenem** (10 µg)	≥ 23	≤ 19	R (0)	R (0)	R (0)	R (0)	R (0)	R (0)	R (0)	R (4)	R (5)	R (6)	R (2)	0%
**Gentamycin**(10 µg)	≥ 15	≤ 12	S (25)	S (23)	S (20)	S (20)	R (0)	S (16)	S (19)	R (8)	R (5)	S (17)	R (5)	63.64%
**Tetracycline** (30 µg)	≥ 15	≤ 11	R (0)	R (0)	R (0)	R (0)	I (12)	R (0)	S (16)	S (18)	S (17)	R (10)	R (1)	27.27%
**Azithromycin** (5 µg)	≥ 13	≤ 11	R (0)	S (15)	R (0)	R (0)	R (0)	R (0)	R (0)	R (11)	R (9)	S (2)	R (4)	18.19%
**Trimethoprim** (5 µg)	≥ 16	≤ 10	R (0)	R (0)	R (0)	S (20)	R (0)	S (20)	S (24)	S (23)	S (23)	R (8)	R (9)	45.55%
**Colistin (**10 µg)	≥ 11	≤ 10	R (9)	R (10)	R (7)	R (8)	R (8)	R (4)	R (8)	R (8)	R (5)	R (0)	R (1)	0%
**Fosfomycin** (10 µg)	≥ 16	≤ 12	R (0)	R (0)	R (0)	R (0)	R (0)	R (0)	R (0)	R (2)	R (2)	R (0)	R (0)	0%
**Chloramphenicol**(30 µg)	≥ 18	≤ 12	S (27)	S (25)	S (20)	R (2)	S (20)	R (0)	S (22)	S (26)	S (30)	S (22)	S (17)	81.82%
**Nitrofurantoin**(300 µg)	≥ 18	≤ 12	I (14)	I (14)	I (14)	R (10)	R (0)	R (0)	R (5)	I (16)	I (16)	R (10)	R (4)	0%
**Ciprofloxacin**(5 µg)	≥ 26	≤ 21	R (18)	R (4)	R (16)	R (10)	R (8)	R (8)	I (22)	R (16)	R (15)	R (13)	R (18)	0%
**MAR INDEX**^d^			**0.75**	**0.67**	**0.67**	**0.83**	**0.83**	**0.83**	**0.58**	**0.67**	**0.67**	**0.75**	**0.92**	

^a^(EM100 connect – CLSI M100 ED30, 2020)

^b^*S, I & R* (Sensitive, Intermediate & Resistant)

^c^*AE* Antibiotic effectiveness % = (No of Sensitive strain/total No of strain)

^d^Multi antibiotics resistant MAR = a/b, where a represents the number of antibiotics to which the test isolate depicted resistance and b represents the total number of antibiotics to which the test isolate has been evaluated for susceptibility

## Antibacterial Activities against Gram-Negative and Gram-Positive Bacteria

A disk diffusion assay [33] examined the antibacterial effects of NPP and MPP against seven tested pathogenic bacteria.

## Minimum inhibitory concentration (MIC)

The minimum inhibitory concentration (MIC) of NPP and MPP was determined using disk diffusion techniques [31], following the procedures of Drew et al. [34]. The MIC of an antimicrobial agent was defined as the lowest concentration (μg/mL), inhibiting the visible growth of a microorganism after 24-48 h.

### Disk diffusion assay

The Kirby-Bauer disk diffusion method [34, 35] was used to investigate NPP and MPP's antibacterial activity against the seven previously mentioned bacteria. Plates containing BHI agar (Oxoid) were inoculated and spread by inocula of about 2 × 10^5^ CFU mL^−1^ for each strain. Then, 5 mm-diameter sterilized paper disks were placed at appropriate distances on the surface of BHI agar medium after soaking in diluted NPP and MPP solutions of the following concentrations: 12.5, 50, 100, 200, 400, 800, and 1000 g/mL for NPP and 0.5, 1, 2, 5, 10, 20, 40, 50, and 100 for MPP. Plates were incubated at 37C° for 24–48 h. Then, a transparent millimeter ruler was used to measure the diameters of the developed zones (mm). The net inhibition zone was then calculated by subtracting the original disks' (5 mm) diameter from the total zone diameters.

## Bacterial growth curve (turbidity test)

Aliquots (50 µL) of the previously mentioned bacterial cultures were taken from the cultures incubated at 37 ◦C for 4 h in BHI broth and dispensed into each well of a 96-well plate. The bacterial cells were treated with 50 µL of 1 MIC of either NPP or MPP. The negative control contained only BHI broth, while the positive control contained cell cultures without further treatment. Growth was measured using a microplate reader (Bio-Rad 680XR, Hertfordshire, U.K.) to asses the turbidity (optical density) (O.D) at 600 nm as an indicator of bacterial growth at 0 h, 6, 12, 18, and 24 h of incubation.

## Comparison of single MPP, Gentamicin treatment and their combination

The experiment followed a disk diffusion assay. Sterilized filter paper disks with a diameter of about 5 mm were saturated with 4 μL of 20 μg/ml concentration of either MPP or Gent in a single treatment. Then, samples were placed on BHI agar plate incubated with 15 μL of tested bacteria suspension. Mixed preparations of 2 µL of both Gent (10 µg/ml) and MPP (10 µg/ml) were used in combination treatments.

## Transmission electron microscope (TEM)

*Staphylococcus pasteuri* (Gram-positive) and *Salmonella* Typhimurium (Gram-negative) were chosen as targets for TEM analysis as they were shown to be highly inhibited by MPP. The bacteria cultivated on brain heart infusion broth were treated with 20 μg/mL of Gent and MPP separately or in combination and then incubated at 37 °C for 4 h before analysis, while the control bacterial suspensions were kept without any treatment. The examination procedures were conducted as previously described by Sitohy [36].

## Statistical analysis

Descriptive measurements of the data were analyzed using the SPSS program version 23. A two-way ANOVA test was conducted to compare various microorganisms (*Staph pasteuri*, *S. pyogenes*, *S*. Typhimurium*, K. pneumonia*, *E. coli*, *K. oxytoca*, and *P. aeruginosa*), different substances (MPP and Gent), and different years (2020–2022), as well as the interactions between them, concerning the diameter of the inhibition zone. The null hypothesis was rejected if the *p*-value was less than 0.05, referring to the significant differences between the means. Conversely, if the p-value was greater than 0.05, it indicated a lack of significant difference.

A post hoc test using Duncan's test was employed to conduct multiple comparisons among the average values of the study groups. Means that shared the same letter were deemed not significantly different at the 5% probability level, as determined by Duncan's multiple range tests.

## Results

### Frequency of bacterial resistance against commonly used antibiotics

The bacterial resistance was estimated in a collection of 16 pathogenic bacterial isolates regrouping 5 Gram-positive and 11 Gram-negative bacteria (Table 1). The antibiotic susceptibility test of Gram-positive bacteria was carried out against 12 commonly used antibiotics, i.e., Ciprofloxacin, Chloramphenicol, Vancomycin, Gentamicin, oxacillin, Azithromycin, Rifampin, Nitrofurantoin, Trimethoprim, Tetracycline, Clindamycin, Linezolid. Also, the susceptibility of Gram-negative bacteria to antibiotics was studied using 12 antibiotics viz Ampicillin–sulbactam, Cefepime, Meropenem, Gentamycin, Tetracycline, Azithromycin, Trimethoprim, Colistin, Fosfomycin, Chloramphenicol, Nitrofurantoin, Ciprofloxacin.

For Gram-positive bacteria, the tested bacterial strains came in the subsequent descending order according to MAR (multi antibiotics resistant index), *S. aureus* (OSC) (0.92 MAR) > *B. cereus* and *S. pyogenes* (0.75 MAR) > *S. pasteuri* and *S. aureus* (0.42 MAR). Considering the antibiotic effectiveness (AE%), only three antibiotics were still effective against the tested Gram-positive bacteria: (Gentamicin, Chloramphenicol, Ciprofloxacin). Gentamicin achieved 80% AE, i.e., out of five tested bacteria, four were susceptible, i.e., *Staph pasteuri*, *Staph aureus*, *Staph aureus* ‘OSC’ and *S. pyogenes*. Chloramphenicol exhibited 60% AE, i.e., against 3 of the 5 tested pathogenic bacteria (*Staph pasteuri, Staph aureus, S. pyogenes*) were susceptible. Likewise, Ciprofloxacin recorded 60% AE against 3 out of the 5 tested pathogenic bacteria (*Staph pasteuri, Staph aureus* and *Bacillus cereus*). One antibiotic (Tetracycline) was weakly effective, recording 40%AE, i.e., it was effective against 2 of 5 tested pathogenic bacteria. It can be concluded that most Gram-positive bacteria were resistant to the antibiotics that were used. The least resistant bacteria were *Staph pasteuri*, *Staph aureus*, showing 0.42 MAR. In contrast, the highest resistant bacteria were (*Bacillus cereus*, *S. pyogenes*, *Staph aureus* ‘OSC’), recording (0.75,0.75,0.92 MAR index), generally meaning that most Gram-positive bacteria were resistant to antibiotics.

For Gram-negative, the MAR index of the tested eleven Gram-negative bacteria showed the highest value (0.92) for *Proteus mirabilis* which could resist eleven antibiotics among the total twelve ones, i.e., the highest resistant. Three strains of (*Klebsiella pneumonia* (1,2,3) recorded the highest second MAR index value (0.83), i.e., they resisted ten antibiotics among the total twelve ones. Two isolates (*E. coli* ^1^, *p. aeruginosa*) recorded the highest third MAR index value (0.75) i.e., they resisted nine antibiotics among the twelve total ones. Four isolates (*E. coli* ^2^, *E. coli* ^3^, *S*. Typhimurium, *Shigella sp*) recorded the highest fourth MAR index value (0.67), i.e., they resisted eight antibiotics among the total twelve ones. One isolate (*K. oxytoca*) recorded a lower MAR index (0.58) and can be considered moderately resistant, i.e., resisting seven antibiotics among the tested ones.

Considering the AE%, only two antibiotics (Chloramphenicol and Gentamycin) were still effective against 9 and 7 of the tested pathogenic bacterial isolates, respectively, achieving 82% and 64% AE, respectively. One antibiotic (Trimethoprim) was weakly effective, recording 45.55% AE. Two antibiotics (Tetracycline and Azithromycin) were ineffective, recording 27.3% and 18.2% AE, respectively. However, most of the antibiotics were mainly ineffective against the tested bacteria. Seven antibiotics (Ampicillin-sulbactam, Cefepime, Meropenem, Colistin, Fosfomycin, Nitrofurantoin, and Ciprofloxacin) were ineffective, recording 0% AE against the whole tested bacterial collection.

It can be concluded that most tested Gram-negative bacteria were resistant to the antibiotics. The least resistant bacteria showed a 0.58 MAR index, i.e., they resisted more than 50% of antibiotics, and the antibiotic resistance varied between the highest and moderate resistance. All gram-negative bacteria (100%) of the total bacteria used showed a MAR index higher than (0.5), so it can be stated that all the gram-negative bacteria developed resistance against all the tested antibiotics. Three Gram-positive bacteria (60%) of the total bacteria used showed a MAR index higher than (0.5), referring to the development of high resistance to most total antibiotics.

Eight antibiotics were totally ineffective achieved AE in the range (0–20%) against the total tested Gram-positive bacteria compared to 9 antibiotics in case of Gram-negative bacteria. Thus, only three antibiotics showed effectiveness against Gram-positive compared to only two against Gram-negative bacteria.

### Chemical characterization of NPP and MPP

#### SDS-PAGE, Urea-PAGE and pH-solubility

**Fig. 1:**
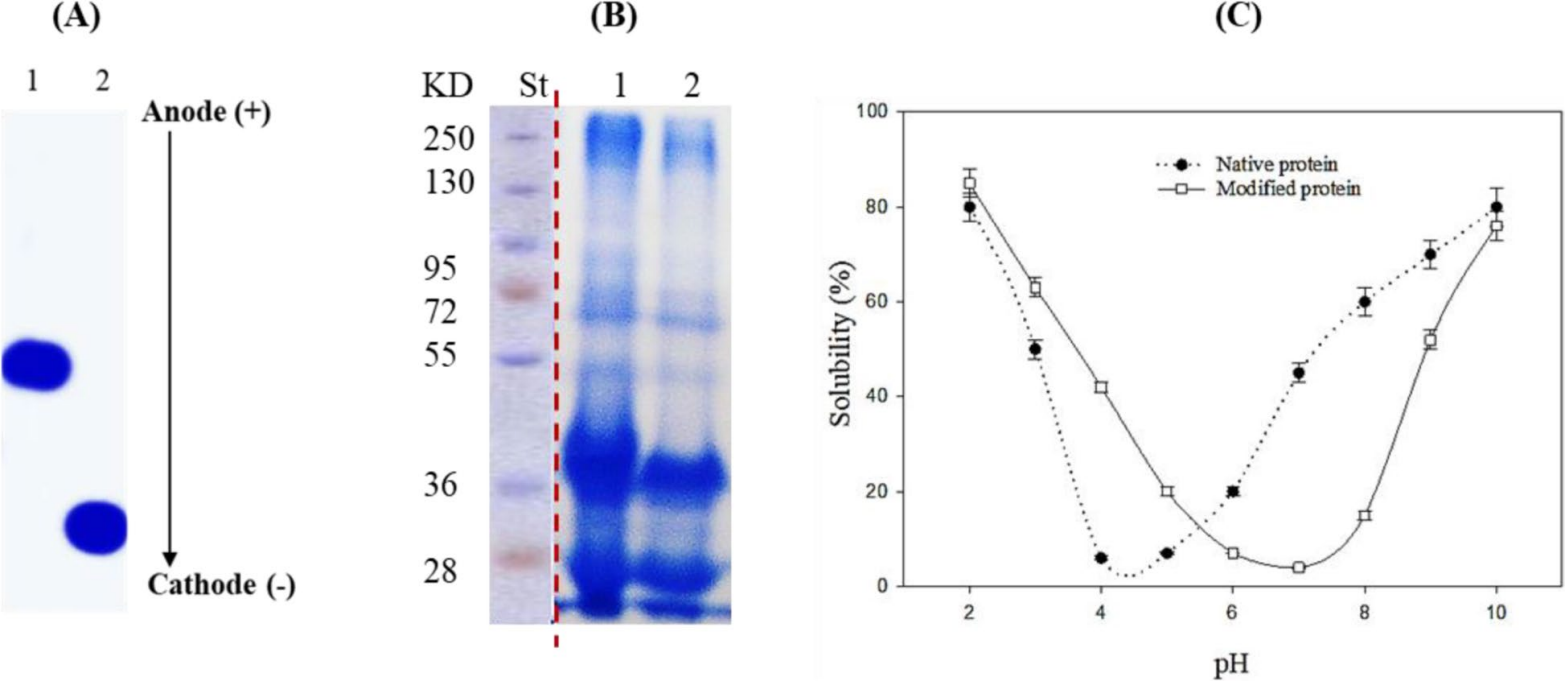
UREA-PAGE electropherogram (**A**), SDS-PAGE (**B**) and pH-solubility curves (**C**) of native (NPP) and methylated (MPP) *Phaseolus vulgaris* seed protein. The first lane in B, representing the molecular weight standard, was taken from another gel run at the same time and conditions and the dotted line separates it from the SDS-PAGE gel of the proteins samples which was cropped from original SDS-gel (shown in the supplementary file)

The data in Fig. 1 present the chemical characteristics of the prepared methylated *Phaseolus vulgaris* seed protein (MPP), as analyzed by UREA-PAGE, SDS-PAGE, and the pH solubility curve and compared to the native protein (NPP). The data revealed a faster migration of MPP towards the cathode than the native protein. However, the SDS-PAGE of MPP was not different from the native protein. The main protein components in the two cases appeared at 28, 36, 55, 72 and 250 kD. The pH solubility curve revealed that the least soluble point of the MPP was at pH 7.1 against pH 4.4 for the NPP.

#### Fourier-Transform Infrared (FT-IR) Spectroscopy

**Fig. 2:**
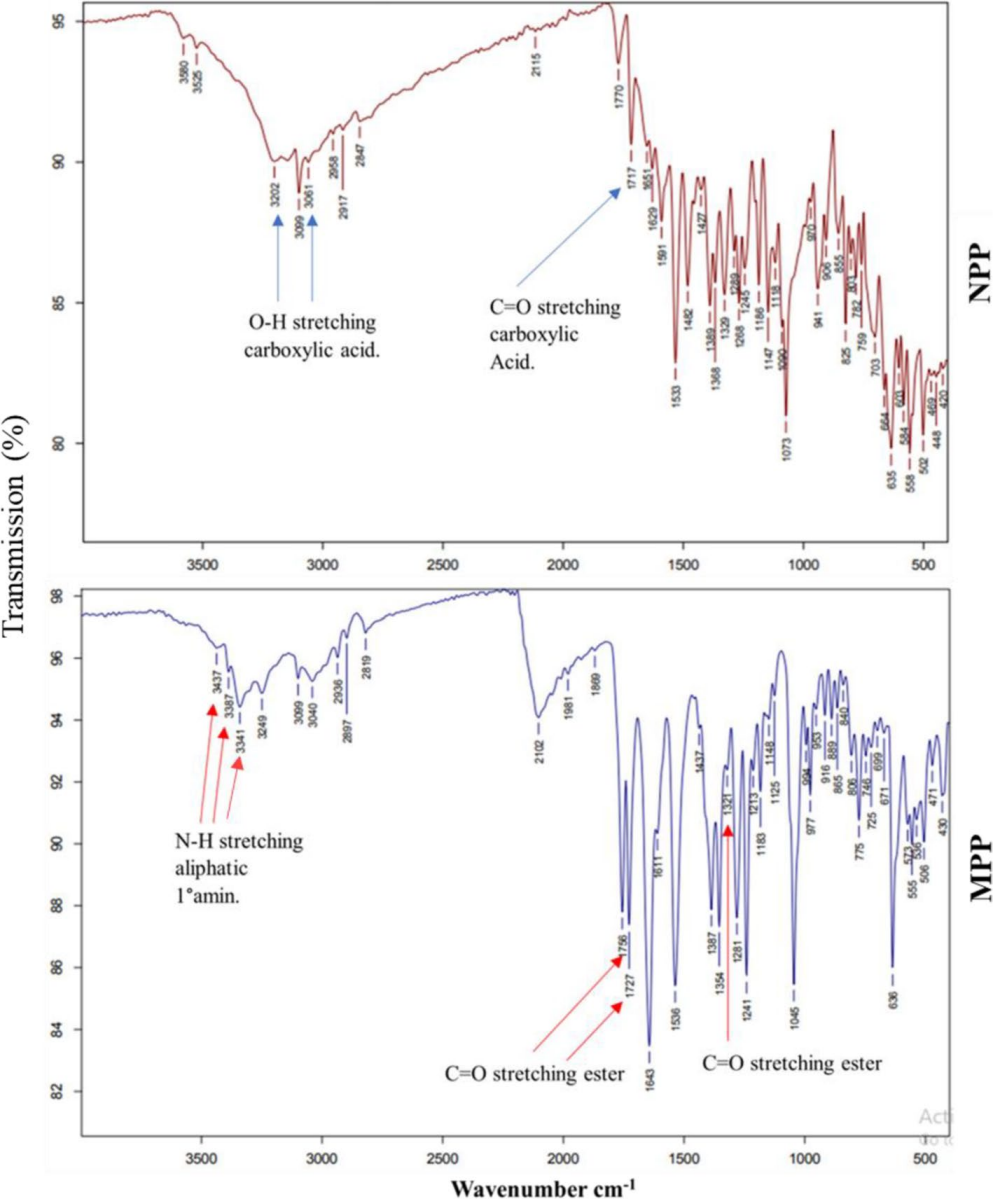
FT-IR spectra of native (NPP) and methylated (MPP) *Phaseolus vulgaris* seed protein. The peaks referring specific functional groups are referred to by arrows in each case

It is evident in Fig. 2 that the modified form of *Phaseolus vulgaris* seed protein (MPP) contains three peaks at wave numbers (3437,3387, 3341 cm-1) referring to the presence of N–H stretching aliphatic primary amine group and three wave numbers (1756,1727,1213 cm-1) referring to C = O stretching ester which were absent from the native form (NPP). Alternatively, the native form (NPP) indicated IR absorption peaks at wave numbers (3202, 3061,1717 cm^−1^), which are absent from the modified form (MPP), referring to the presence of O–H stretching carboxylic acid, O–H stretching carboxylic acid and C = O carboxylic acid group which evidenced the presence of the free carboxylic groups. These IR absorption patterns confirmed that the esterification reaction occurred in the carboxyl-free groups, evidently coming from the aspartic and glutamic acid residues. Furthermore, it confirms the establishment of the esterified groups in the modified form. The similarity for the other absorption peaks indicates that the modification reaction was only restricted to the regions rich in free carboxylic groups.

### Susceptibility of pathogenic bacteria to NPP and MPP

#### Minimum Inhibitory Concentration (MIC):

**Fig. 3:**
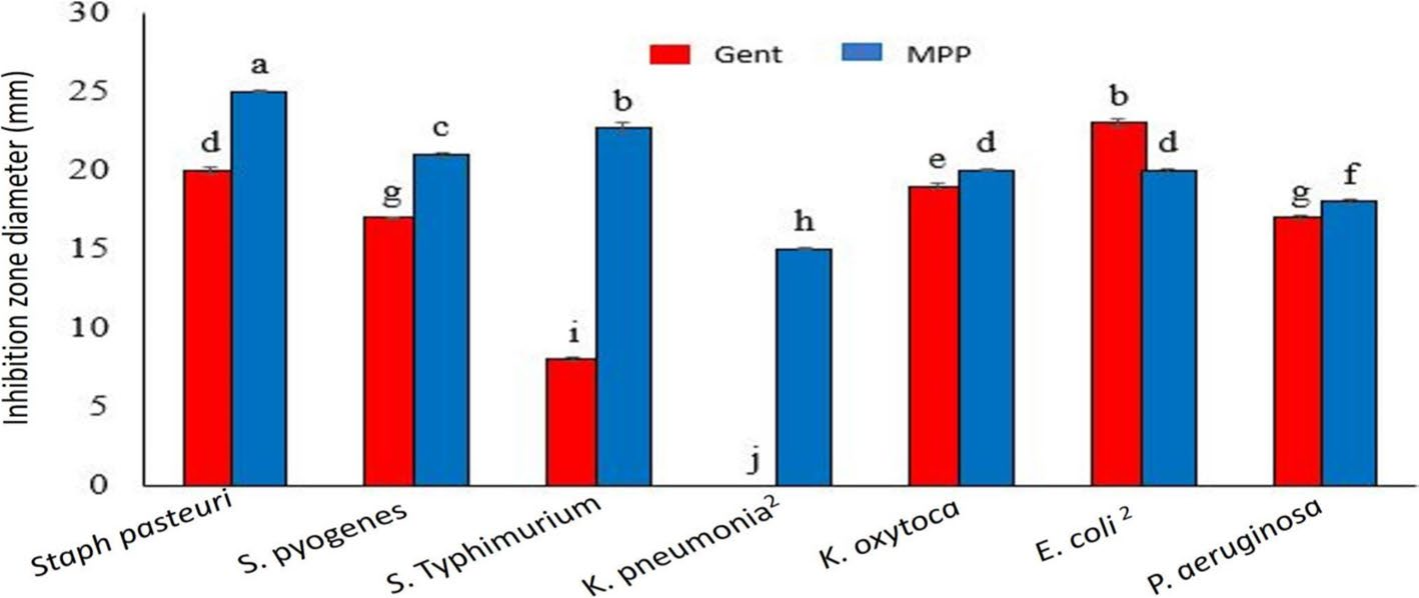
Graphical representation comparing the antimicrobial activity of gentamicin and MPP (methylated Phaseolus vulgaris ) against seven pathogenic bacteria; two Gram-positive and five Gram-negative

Figure 3s illustrates the antibacterial effect of NPP and MPP-graded concentrations against the seven tested pathogenic bacteria. NPP recorded MIC values within the range of 25–200 µg/mL, while MPP produced relatively lower MICs values ranging from 1 to 20 µg/ml as presented in Table 4. Generally, the diameter of the inhibition zone induced by MPP was bigger than that produced by NPP (data not shown) at the same concentration in most cases. MIC of MPP was much less than the MIC of NPP; therefore, MPP with low MIC scores may indicate that less methylated protein is needed to inhibit the growth of the microorganisms. Thus, MPP is more effective as an antimicrobial agent than NPP.

**Table 4:** Minimum inhibitory concentration (MICs; µg/mL) of native and modified protein derived fro*m Phaseolus vulgaris* seed

**Tested bacteria**	Minimum inhibitory concentration (MICs; µg/mL)	
	NPP	MPP
***Staph pasteuri***	100	5
***S. pyogenes***	50	10
***S.* Typhimurium**	50	5
***K. pneumoniae ***^***2***^	100	20
***K. oxytoca***	25	10
***E. coli ***^***2***^	25	1
***p. aeruginosa***	200	2

#### Antibacterial activity of mixed combinations of modified *Phaseolus vulgaris* (MPP) and gentamicin (Gent)

The potential effect of combined preparations of both antibiotic and methylated *Phaseolus vulgaris* seed protein (MPP) was tested by mixing equal volumes of gentamicin (2µL; 10 µg/ml) and MPP (2µL; 10 µg/ml) and subjecting by disk diffusion assay. The combination was compared to the single dose of each (4 µL and 20 µg/ml, respectively). Results are given in Table 5.

**Table 5:** The synergistic effect between gentamicin and the methylated *Phaseolus vulgaris* seed protein (MPP) on different pathogenic bacteria

Microorganism	Inhibition zone diameter (mm)			Theoretical^c^	Microorganism effect
	Gent^a^ (20 µg/ml)	MPP^b^ (20 µg/ml)	MPP + Gent (10 + 10 µg/ml)	G + M (10 + 10 µg/ml)	
***Staph pasteuri***	8.0 j ± 0.22	14.0f ± 0.08	18.9 b ± 0.14	11	13.6 b ± 4.49
***S. pyogenes***	2.5 m ± 0.16	17.0 d ± 0.16	20.0 a ± 0.22	9.75	13.2 c ± 7.66
***S.* Typhimurium**	8.0 j ± 0.08	16.0 e ± 0.45	19.0 b ± 0.43	12	14.3 a ± 4.65
***K. pneumoniae ***^***2***^	3.0 m ± 0.22	10.0 h ± 0.16	9.0 i ± 0.41	6.5	7.3 f ± 3.13
***K. oxytoca***	4.0 l ± 0.82	16.0 e ± 0.08	16.0 e ± 0.17	10	12.0 d ± 5.67
***E. coli ***^***2***^	4.0 l ± 0.82	12.0 g ± 0.36	14.0 f ± 0.82	8	10.0 e ± 4.35
***P. aeruginosa***	5.0 k ± 0.37	16.0 e ± 0.41	18.0 c ± 0.08	10.5	13.0 c ± 5.72
Antibacterial effect	4.9 c ± 2.08	14.4 b ± 2.42	16.4 a ± 3.61		

^a^*Gent* Gentamicin

^b^MPP Modified *Phaseolus vulgaris* protein

^c^Theoretical G + M: The calculated value of the inhibition zone induced by the two single **(**10 + 10 µg/ml) treatments each of antibiotic and MPP against tested bacteria

For the Gram-positive bacteria *Staphylococcus pasteuri* [23], the experimental combination treatment produced a 19 mm inhibition zone diameter. The Theoretical inhibition zone of the two single treatments of antibiotic and MPP is equal to 11 mm (4 + 8 mm), which is inferior to the experimental combined treatment. This observed increase in the experimental treatment might be due to specific synergetic effects.

Likewise, the calculated value of the inhibition zone inducible by the two single treatments, antibiotic and MPP against *Streptococcus pyogenes* (LMG21599), is equal to 9.75 mm (1.25 + 8.5 mm), i.e., it is inferior to the experimental combined treatment (20 mm) referring to a synergetic effect. Similar results occurred with the Gram -negative bacteria *Salmonella* Typhimurium, *Klebsiella pneumoniae*^2^, *Klebsiella oxytoca, E. coli*^2^, *Pseudomonas aeruginosa*. The theoretically calculated values were 12, 6.5, 10, 8, 10.5 mm against 19, 9, 16, 14, 18 mm for the experimental combined treatments, respectively. The differences between the theoretical and experimental values confirm the presence of synergetic effects in all cases.

#### Changes in bacterial susceptibility to Gentamycin and MPP across 2 years

Testing the antimicrobial activity of MPP and Gent two years after the first measurements revealed that Gent's inhibition zone diameters decreased, especially in the case of *K. pneumoniae*2 and *K. oxytoca*, in contrast to MPP, which showed nearly the same diameter of the inhibition zone. This result means that the long storage time of MPP has no or little effect on its activity, and the bacteria could not develop resistance against MPP, the opposite of the case with the antibiotic Table 6.

**Table 6:** Antimicrobial activity of gentamicin and modified *Phaseolus vulgaris* protein across long time

Bacterial Strains	Inhibition zone diameter (mm)			
	Gent (20 µg/ml)		MPP (20 µg/ml)	
	2020	2022	2020	2022
***Staph pasteuri***	10.00 g ± 0.06	8.00 I ± 0.15	14.00 c ± 0.21	14.00 c ± 0.06
***S. pyogenes***	4.00 k ± 0.10	2.00 m ± 0.06	17.00 a ± 0.12	17.00 a ± 0.06
***S.* Typhimurium**	10.00 g ± 0.06	8.00 I ± 0.12	16.00 b ± 0.15	16.00 b ± 0.06
***K. pneumoniae ***^***2***^	11.00 f ± 0.15	3.00 l ± 0.06	10.00 g ± 0.06	10.00 g ± 0.12
***K. oxytoca***	13.00 d ± 0.15	4.00 k ± 0.06	13.00 d ± 0.06	16.00 b ± 0.25
***E. coli ***^***2***^	9.00 h ± 0.15	4.00 k ± 0.17	14.00 c ± 0.12	12.00 e ± 0.15
***P. aeruginosa***	5.00 j ± 0.15	5.00 j ± 0.06	16.00 b ± 0.12	16.00 b ± 0.15

#### Comparison between bacterial susceptibility to Gentamycin and MPP

Comparing the results of gentamicin disks from antibiotics susceptibility tests at 10 µg with MPP results from MIC test at 10 µg, it showed more effectiveness in the second case than the first case Fig. 3. MPP was effective against all seven tested bacteria, while gentamicin was only effective against five (Table 1s). Gentamicin disks induced low or no inhibition zone diameters (8 mm and 0 mm, respectively) against the Gram-negative strains (*Salmonella* Typhimurium, *K. pneumoniae*2), indicating total bacterial resistance based according to the Clinical and Laboratory Standards Institute [32]. On the contrary, MPP disks achieved inhibition of more extensive zone diameters (22 mm and 15 mm) against these two strains, respectively, indicating evident bacterial susceptibility.

#### Bacteria growth curve in the presence of NPP and MPP

**Fig. 4:**
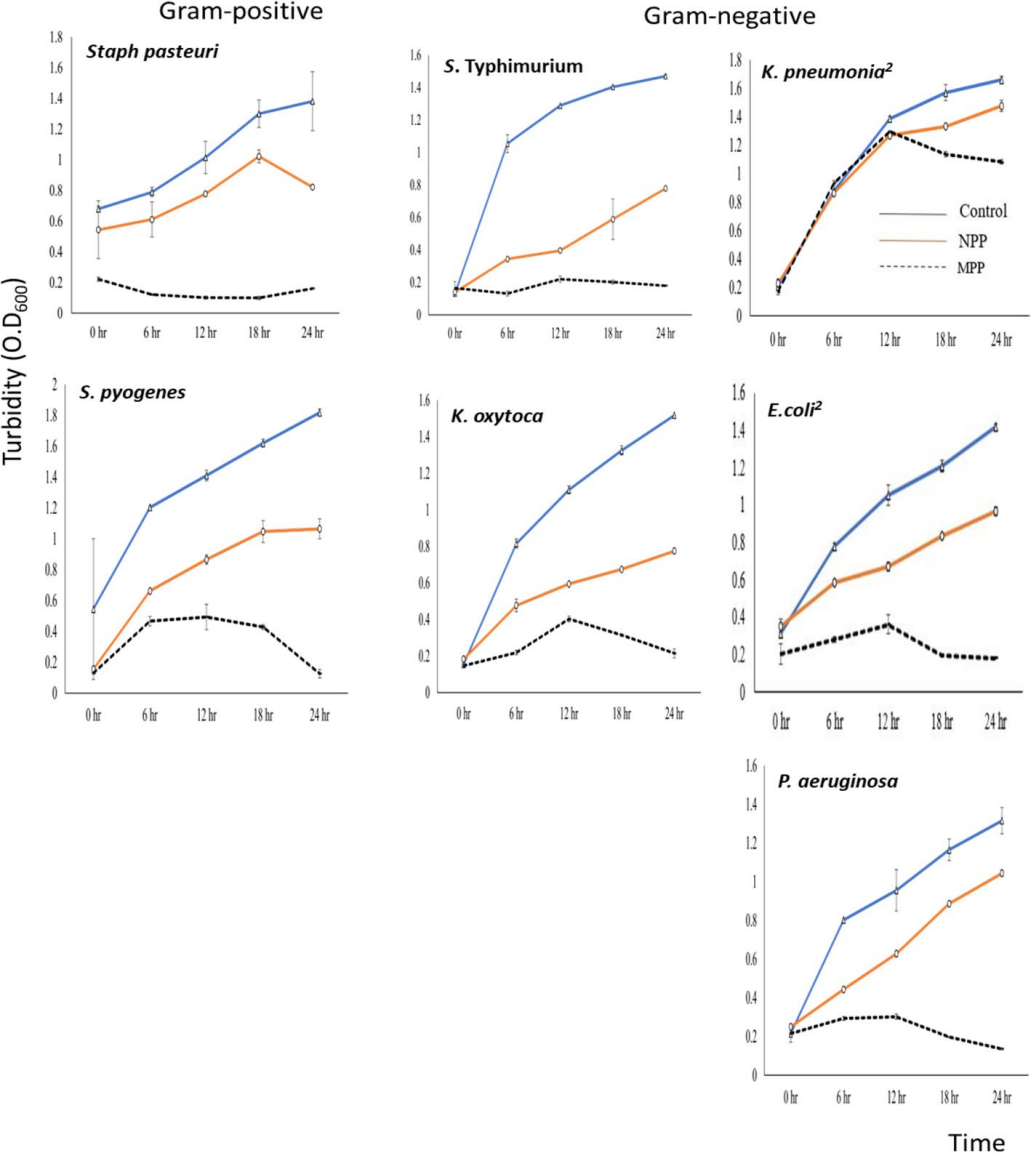
Twenty-four-hours growth curves of seven pathogenic bacteria exposed to one MIC of NPP and MPP, compared to untreated control, as a function of the bacterial turbidity measure at 600 nm

Figure 4 shows that the 24 h growth curves of seven pathogenic bacteria exposed to one MIC for either NPP or MPP generally indicated growth inhibition. The growth of tested control bacteria reached the maximum OD value (1.0 -1.8) within almost 12–24 h. The presence of 1.0 MIC of MPP and NPP prevented the bacteria from reaching this OD maximum. The growth of the Gram-positive bacteria, *Staph pasteuri* and *Streptococcus pyogenes*, was moderately inhibited by NPP (48.75% and 41.42%), respectively, but maximally inhibited by MPP (93.3% and 93.0%) after 24 h of incubation at 37 C°. The presence of NPP and MPP inhibited the growth of all the Gram-negative bacteria. These two antibacterial agents (NPP and MPP) inhibited the growth of four Gram-negative bacteria (*Salmonella* Typhimurium*, K. oxytoca*, *E. coli*^*2*^ and *P. aeruginosa*) by about (47.03%—87.76%), (48.96% -85.84%), (31.5%—86.9%) and (20.67%-86.16%), respectively after 24 h at 37 C°. However, *K. pneumoniae*^2^ bacterium was the least susceptible to the natural proteins, recording a low level of inhibition, i.e., corresponding to 11.05% and 34.72% in case NPP and MPP, respectively.

#### TEM image analysis

**Fig. 5:**
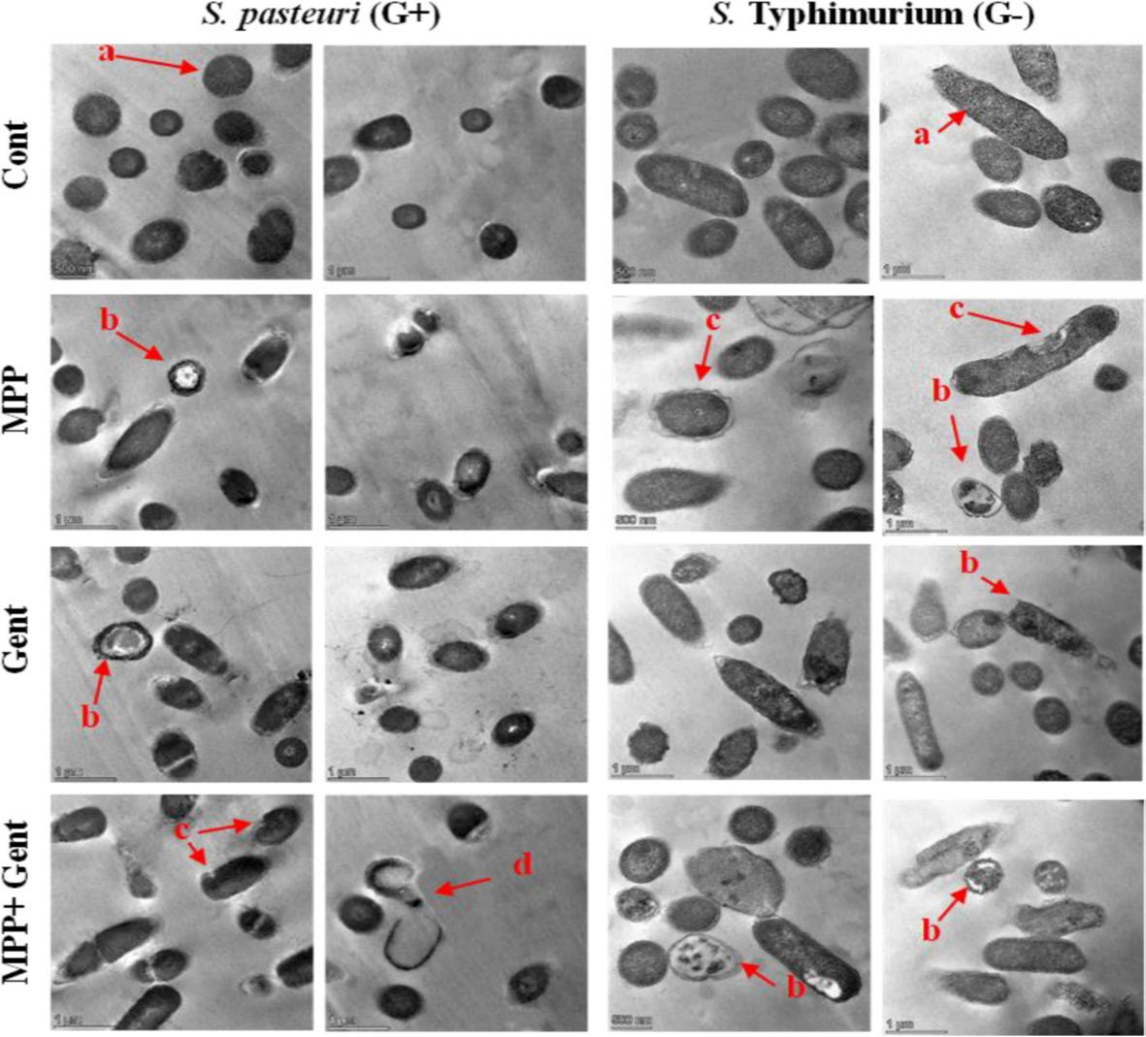
Transmission electron microscopic (TEM) images (11.000X.) of *Staphylococcus. pasteuri* (Gram-positive) and *Salmonella* Typhimurium (Gram-negative) as affected by 20 µg/mL of MPP, gentamicin (Gent) and combination of MPP + Gent as compared to control (Cont). Two images were taken at the same magnification power for each treatment. The small letters of a, b, c, and d refer to normal bacterial cells, cell components lysis, cell wall destroy and completely deformed bacterial cells, respectively

The Gram-positive bacterium (*S. pasteuri*) and the Gram-negative bacterium *(S*. Typhimurium) were grown in BHI broth media treated with (20 µg/mL) of MPP or Gentamicin separately and in combination (10 + 10 µg/mL. TEM images of the bacterial cells showed various deformation processes after 4 h of incubation at 37◦C (Fig. 5). The single MPP treatment led to visible cell wall raptures, vacuoles lysis, cell swelling, cell shrinkage, and reduced viable cells. The single antibiotic treatment (Gent) induced signs of cell division and separation, cell lysis,, and lysis of cell components. However, the number of cells was higher than those treated with MPP, and cell wall rapture was nearly absent. The combination between MPP and Gent induced more deformation signs regrouping those of the single treatments, i.e., increased cell components lysis, bacterial cell loss, swelling and shrinkages, and cell separation and division, particularly in Gram-positive bacterium.

## Discussion

This study aimed to test the native (NPP) and methylated (MPP) *Phaseolus vulgaris* seed proteins individually or in combination with the antibiotic Gentamicin against some multidrug-resistant bacteria. MPP proved more effective than NPP. Combining MPP with Gentamicin further enhanced its antibacterial activity through its different modes of action. Three Gram-positive bacteria (60% of the total used bacteria) showed MAR index higher than 0.5, referring to highly developed resistance to most tested antibiotics. Moreover, all the tested Gram-negative bacteria (100% of the total tested ones) also showed a MAR index higher than 0.5, i.e., they may have developed resistance against most tested antibiotics. Therefore, it can be concluded that most of the tested Gram-positive or Gram-negative bacteria developed multidrug resistance against the commonly used antibiotics. Since the 12 antibiotics used in the current study belong to the principal five mechanism-based antibiotic groups (cell wall synthesis, protein synthesis, DNA synthesis, RNA synthesis and folic acid synthesis), the shown developed resistance may represent a big challenge. Multidrug-resistant bacterial strains may have developed because of excessive and inadequate use of antibiotics in human and veterinary medicine [37]. The outer membrane of gram-negative bacteria is the primary cause of resistance to a wide range of antibiotics, including lactams, quinilons, colistins, and others [38]. Gram-negative bacteria have this essential layer, making them more antibiotic-resistant than Gram-positive bacteria [39–41] explaining the spread of drug resistance in 100% of the tested Gram-negative bacteria.

Eight antibiotics proved ineffective against the tested Gram-positive bacteria compared to 9 antibiotics against the Gram-negative bacteria, i.e., most tested antibiotics were ineffective against most Gram-positive and Gram-negative bacteria. Only two antibiotics (Gentamicin -Chloramphenicol) were still highly effective against Gram-positive and Gram-negative bacteria, achieving antibiotic effectiveness (AE) in the 64–80% range. The high antibiotic action of Gentamycin is associated with its mode of action based on its binding to the 16 s rRNA at the 30 s ribosomal subunit, disturbing mRNA translation and leading to non-functional proteins [42]. Thus, it is less probable to form Gentamycin-resistant bacteria.

Thus, discovering new antimicrobial agents with novel mechanisms of action is critically needed [43]. Natural alternatives may have much higher potential. Alkaline proteins, herbs, spices, and herbal extracts have been reported for their antibacterial effects [44]. Within this scope, the native protein (NPP) was isolated from *Phaseolus vulgaris* seed before its methylation into MPP to enhance its antimicrobial activity. Chemical characterization confirmed the intended structural changes. The speeder migration of MPP towards the cathode than the native protein (NPP) is due to the enhanced positive charges on the modified protein by the esterification process following revealing [45]. The similarity of the SDS-PAGE of MPP and NPP, showing five main protein bands at 28, 36, 55, 72 and 250 kD, may be a result of the fact that the grafted CH_3_ groups during esterification do not induce a significant change in the molecular mass because of its low mass. The pH solubility curve of MPP and NPP revealed the least protein solubility points at pH 7.1 and 4.4, indicating considerably a higher isoelectric point in MPP due to the higher net positive charge enhanced by the esterification process in accordance with [45]. This higher positive charge and isoelectric point are essential for the antibacterial potential activity of the protein following [45]. The different IR absorption patterns of NPP and MPP evidenced the transformation of the free carboxylic groups coming from aspartic and glutamic acid on the protein molecule into the methylated carboxylate group. MPP absorption peaks exhibited signals referring to the ester groups, while NPP produced signals indicating the free carboxyl groups [45].

The MIC range of MPP was 10–25 times lower than NPP. The high antimicrobial activity of MPP could be due to their relative richness in alkalinity and hydrophobicity, initiating the electrostatic interactions between the antimicrobial agent’s positive charges and the bacterial cell walls or membranes’ negative charges. This conclusion supports a previous study on the influence of positive charge, amplified by the methylation process, on the effectiveness of antibacterial agents. Alternatively, MPP exhibited antimicrobial activity higher than or nearly matching the most effective antibiotic (gentamicin) as both have MIC in the range (1–20 µg/ml) against the studied Gram-positive and Gram-negative bacteria. However, this practical range of MIC is relatively lower than 11S and M11S derived from other legume proteins, such as lupine seed 11S proteins, where the relevant MIC was in the range of 0.1–2 µg/ml [16]. This relatively low range of MIC is apparently due to the high positive charge of MPP. The broader inhibition of pathogenic bacteria could be due to the synergetic effect of the combined treatment between gentamycin and MPP, resulting from different mechanisms of antibacterial inhibitory action, targeting the 16 s rRNA at the 30 s ribosomal subunit in the first case, while targeting the cell membrane in the second case. This variation makes the inhibitory action additive and not competitive. The more potent antibacterial activity of the combined antibiotic-protein treatment agrees with previously published research [8]. Synergism might have occurred as Gent and MPP may contain polar and non-polar chemical residues. Thus, polar residues can form hydrogen bonds with non-polar residues allowing their synergism [46]. This combined action between Gent and MPP can be the base of new therapeutic techniques involving less antibiotic quantity.

The observed deterioration in the antimicrobial activity of Gent kept in the closed original packages under cold storage (8–10 °C) after two years from the first measurement, especially against the Gram-negative bacteria, in contrast to MPP, may indicate higher keeping quality of MPP than the antibiotic. Alternatively, the pathogenic bacteria could have developed drug resistance against the antibiotic and not against the natural protein derivative. The enhanced resistance of the bacteria towards Gentamycin may be due to enzymatically modified and inactivated aminoglycosides, reduced permeability, and modified 30S ribosomal subunit that interferes with the aminoglycoside binding [42].

The 24 h growth curves of six pathogenic bacteria, Gram-positive and Gram-negative, exposed to 1.0 MIC of MPP generally indicated more significant growth inhibition than NPP, ca. 93 and 86.5%, respectively. In contrast, one bacterium (*K. pneumoniae*^2^) showed the least inhibitory action (34.72%). The six bacteria were generally susceptible to the MPP action on their cellular membranes. However, the lower effectiveness of MPP toward the Gram-negative bacteria *K. pneumoniae* may be due to a thicker cellular membrane [47]. This particular isolate was also shown to have the relatively highest MAR index, indicating its general resistance to antibiotics and probably to antimicrobial agents. Moreover, this bacterium was found to be more resistant to Gentamycin after two years of solid media maintenance while regenerating every two weeks.

Subjecting the Gram-positive bacterium (*S. pasteuri*) and the Gram-negative bacterium (*S.* Typhimurium) to 20 µg/mL of MPP or Gentamicin separately and in combination (10 + 10 µg/mL) produced various signs of deformation on the bacterial cells escaping death after four h of incubation at 37◦C. The difference in the mechanism of bacterial inhibitory action between MPP and Gentamycin was associated with different signs of cellular deformation.

While MPP treatment led to visible cell wall raptures, vacuole lysis, cell swelling, cell shrinkage, and reduced viable cells, Gent produced signs of cell division and separation, cell lysis, and lysis of cell components. Moreover, the higher number of surviving viable cells in the case of Gent than MPP refers to more effective inhibitory action in the second case. Cell lysis may result from electrostatic interactions with the positively charged MPP via their negatively charged cell membrane layers arising from teichoic acid and phospholipids [3].

## Conclusions

Most tested bacteria, either Gram-positive or Gram-negative, developed multidrug resistance against the commonly used antibiotics. Gram-negative bacteria are more antibiotic-resistant than Gram-positive bacteria. Most tested antibiotics were ineffective against Gram-positive and Gram-negative bacteria, particularly the second type. Only two antibiotics (gentamicin -chloramphenicol) were still highly effective against Gram-positive and Gram-negative bacteria, achieving antibiotic effectiveness (AE) in the 64–80% range.

The native protein (NPP) isolated from *Phaseolus vulgaris* was methylated into MPP to improve its antimicrobial activity. Chemical characterization confirmed the intended structural changes, particularly the acquired positive charge on the MPP and its enhanced isoelectric point at pH 7.1. This higher positive charge and isoelectric point are essential for the antibacterial potential activity of the protein. The chemical modification has augmented the antibacterial activity of the protein as the MIC range of MPP was 10–25 times lower than NPP due to their relative richness in alkalinity and hydrophobicity initiating the electrostatic interactions between the positive charges on the antimicrobial agent and the negative charges on the bacterial cell walls or membranes. MPP exhibited antimicrobial activity higher than or nearly the same as the effective antibiotic (gentamicin) against the tested Gram-positive and Gram-negative bacteria. There was a synergetic effect when combining gentamycin and MPP, probably due to different mechanisms of antibacterial inhibitory action. The TEM technique further confirmed this effect.

The antimicrobial activity of Gent kept in the closed original packages under cold storage deteriorated after two years of storage, while MPP conserved its activity. During storage, the bacteria might have developed drug resistance against gentamycin but not MPP. MPP (1.0 MIC) could considerably inhibit the 24 h liquid growth curves of six pathogenic bacteria, Gram-positive and Gram-negative, and only one bacterium (*K. pneumoniae*^*2*^) was weakly inhibited, probably due to a thicker cellular membrane. The TEM technique revealed different modes of action between MPP and Gent on the treated bacteria (Gram-positive and Gram-negative) and higher antibacterial activity of MPP than Gent.

### Supplementary Information

**Additional file 1: Figure 1S**.  Antibiotic susceptibility tests (AST) against Gram positive bacteria, the source data of Table 1. **Figure 2S**. Antibiotic susceptibility tests (AST) against Gram negative bacteria, the source data of Table 2. **Figure 3S**. (A) MIC of native Phaseolus vulgaris protein at graded concentration against Gram positive and Gram-negative bacteria (B) MIC of modified Phaseolus vulgaris protein at degraded concentration against Gram positive and Gram-negative bacteria. **Figure 4S.** Graphical representation of the data in Table 4 (The synergistic effect between gentamicin and the methylated Phaseolus vulgaris seed protein (MPP) on different pathogenic bacteria. **Table 1S.**Comparison between antimicrobial activity of gentamicin and MPP.

## Data Availability

Data will be made available on request.

## Abbreviations

AST: Antibiotics susceptibility test
MDR: Multidrug-resistant bacteria
MIC: Minimum inhibitory concentration
NPP: Native *Phaseolus vulgaris* protein
MPP: Modified *Phaseolus vulgaris* protein
BHI: Brain Heart infusion
CLSI: The Clinical and Laboratory Standards Institute
TEM: The transmission electron microscope
MAR: Multi-antibiotic resistant
OSC: Our strain collection
AE%: Antibiotic effectiveness percentage
SDS-PAGE: Sodium Dodecyl Sulfate–Polyacrylamide Gel Electrophoresis
FT-IR: Fourier-Transform Infrared Spectroscopy
Gent: Gentamicin
