# Supplementary material for: Mapping pathogenic bacteria resistance against common antibiotics and their potential susceptibility to methylated white kidney bean protein
Source: BMC Microbiol. 2024 Feb 5;24:49. doi: 10.1186/s12866-024-03202-x (PMC10840264; doi:10.1186/s12866-024-03202-x)
Supplement: Supplementary file 1 — Additional file 1: Figure 1S. Antibiotic susceptibility tests (AST) against Gram positive bacteria, the source data of Table 1. Figure 2S. Antibiotic susceptibility tests (AST) against Gram negative bacteria, the source data of Table 2. Figure 3S. (A) MIC of native Phaseolus vulgaris protein at graded concentration against Gram positive and Gram-negative bacteria (B) MIC of modified Phaseolus vulgaris protein at degraded concentration against Gram positive and Gram-negative bacteria. Figure 4S. Graphical representation of the data in Table 4 (The synergistic effect between gentamicin and the methylated Phaseolus vulgaris seed protein (MPP) on different pathogenic bacteria. Table 1S.Comparison between antimicrobial activity of gentamicin and MPP. [file 12866_2024_3202_MOESM1_ESM.pdf]

Figure 1s

Staph pasteuri

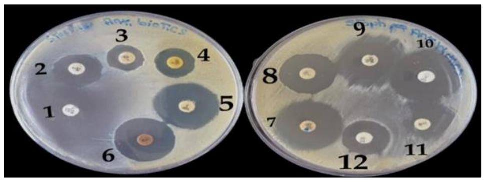

Antibiotics order:
1(LZD30) **2**(DA-2) **3**(RA-5) **4**(F300) **5**(TMP5) **6**(TE-30) **7**(CN120) **8**(OX-1) **9**(AZM15) **10**(CIP5) **11**(C30) **12**(VA-30)

Staph aureus

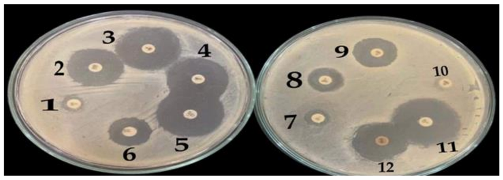

Antibiotics order:
1(DA-2)**2**(CN120) **3**(RA-5) **4**(C30) **5**(LZD-30) **6**(VA-30) **7**(OX-1) **8**(TMP) **9**(F300) **10**(AZM-15) **11**(CIP5) **12** (TE-30)

S. aureus (OSC)

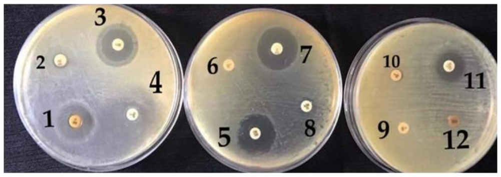

Antibiotics order:
1(F300) **2**(VA-30) **3**(C30) **4**(TMP5) **5**(CN120) **6**(OX-1) **7**(CIP5) **8**(AZM5) **9**(DA-2) **10**(RA-5) **11**(LZD) **12**(TE-30)

Bacillus cereus

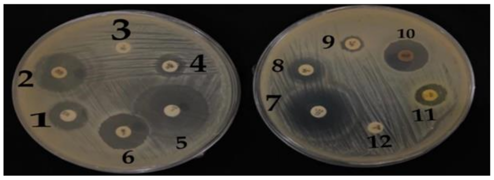

Antibiotics order:
1(VA30) **2**(CN120) **3**(OX-1) **4**(AZM-15) **5**(CIP 5) **6**(C30) **7**(LZD30) **8**(DA-2) **9**(RA5) **10**(TE30) **11**(F300) **12**(TMP5)

S. pyogenes

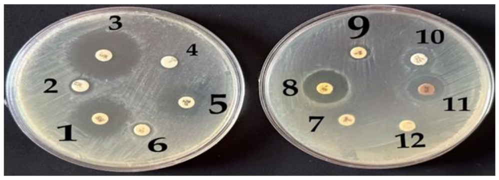

Antibiotics order:
1(CN120) **2**(VA30) **3**(C30) **4**(CIP5) **5**(AZM15) **6**(OX-1) **7**(TMP5) **8**(F300) **9**(RA-5) **10**(LZD30) **11**(TE30) **12**(DA-2)

The 12 used antibiotics:

Ciprofloxacin (CIP5)
Vancomycin (VA-30)
Oxacillin (OX-1)
Rifampin (RA-5)
Trimethoprim (TMP5)
Clindamycin (DA-2)
Chloramphenicol (C-30)
Gentamicin (CN120)
Azithromycin (AZM-5)
Nitrofurantoin (F300)
Tetracycline (TE-30)
Linezolid (LZD-30)

Figure 2s

***E. coli*[1]**

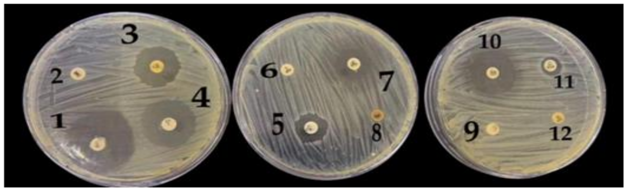

Antibiotics order:
1(C30) **2**(FF-50) **3**(F300) **4**(CIP5) **5**(CIM-10) **6**(TMP5) **7**(AZM15) **8**(TE-30) **9**(A/S20) **10**(CN120) **11**(MEM-10) **12**(FEP30)

***E. coli*[2]**

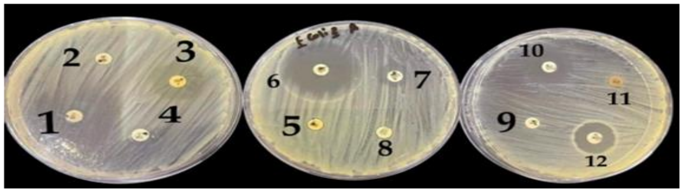

Antibiotics order:
1(C30) **2**(FF50) **3**(F300) **4**(CIP5) **5**(FEP30) **6**(CN120) **7**(MEM-10) **8**(A/S20) **9**(TMP5) **10**(AZM15) **11**(TE-30) **12**(CLM-10)

***E. coli*[3]**

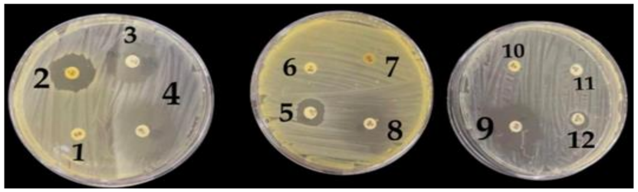

Antibiotics order:
1(FF50) **2**(F300) **3**(CIP5) **4**(C30) **5**(CLM-10) **6**(TMP5) **7**(TE30) **8**(AZM5) **9**(CN120) **10**(FEP30) **11**(A/S20) **12**(MEM-10)

***K. pneumoniae*[1]**

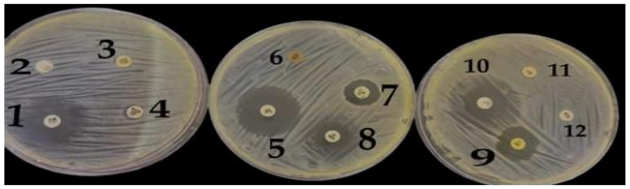

Antibiotics order:
1(CN120) **2**(A/S120) **3**(FEP-30) **4**(MEM-10) **5**(TMP-5) **6**(TE-30) **7**(CLM-10) **8**(AZM15) **9**(F300) **10**(CIP5) **11**(C30) **12**(FF-50)

***K. pneumoniae*[2]**

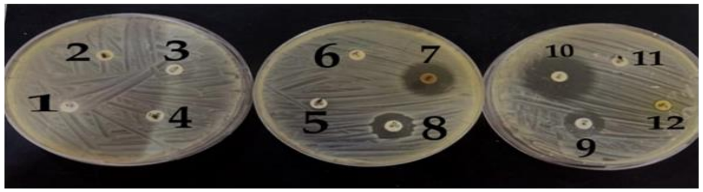

Antibiotics order:
1(A/S120) **2**(FEP30) **3**(CN120) **4**(MEM-10) **5**(AZM15) **6**(TMP5) **7**(TE-30) **8**(CLM-10) **9**(CIP5) **10**(C30) **11**(FF-50) **12**(F300)

***K. pneumoniae*[3]**

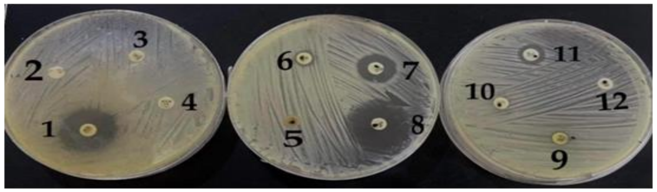

Antibiotics order:
1(CN120) **2**(A/S120) **3**(AZM15) **4**(MEM-10) **5**(TE-30) **6**(FEP30) **7**(CLM-10) **8**(TMP-5) **9**(F300) **10**(C30) **11**(CIP-5) **12**(FF-50)

K. oxytoca

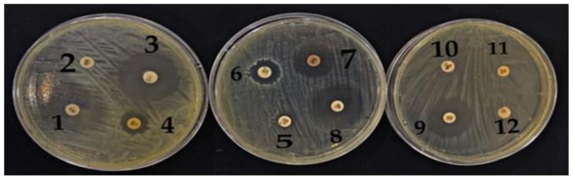

Antibiotics order:
1(C30) **2**(FF-50) **3**(CIP-5) **4**(F300) **5**(AZM-15) **6**(CLM10) **7**(TE-30) **8**(TMP5) **9**(CN120) **10**(MEM-10) **11**(FEP30) **12**(A/S20)

***S.* Typhimurium**

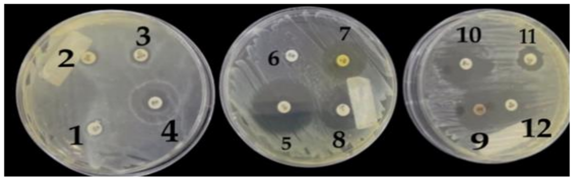

Antibiotics order:
1(A/S20) **2**(FEP30) **3**(MEM-10) **4**(CN120) **5**(C30) **6**(FF-50) **7**(F300) **8**(CIP-5) **9**(TE-30) **10**(TMP5) **11**(CLM10) **12**(AZM-15)

Shigella sp

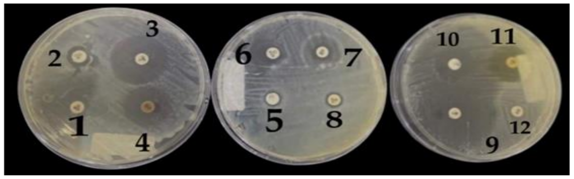

Antibiotics order:
1(AZM-15) **2**(CLM10) **3**(TMP5) **4**(TE-30) **5**(A/S20) **6**(MEM-10) **7**(CN120) **8**(FEP30) **9**(C30) **10**(CIP-5) **11**(F300) **12**(FF-50)

Proteus.mirabilis

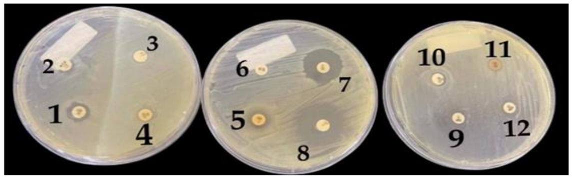

Antibiotics order:
1(CN120) **2**(MEM-10) **3**(A/S20) **4**(FEP30) **5**(F300) **6**(FF-50) **7**(C30) **8**(CIP-5) **9**(TMP5)**10**(CLM10) **11**(TE-30) **12**(AZM-15)

P. aeruginosa

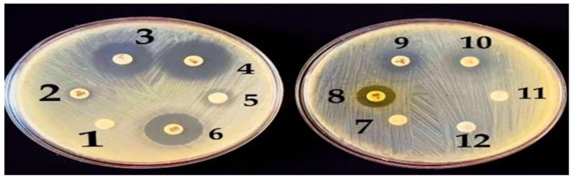

Antibiotics order:
1(A/S20) **2**(AZM-15) **3**(CIP-5) **4**(C30) **5**(MEM-10) **6**(CN120) **7**(FEP-30) **8**(F300) **9**(TMP5) **10**(TE-30) **11**(CLM10) **12**(FF-50)

The 12 used antibiotics:

**Ciprofloxacin** (CIP5)	**Chloramphenicol** (C-30)
**Fosfomycin** (FF-50)	**Gentamicin** (CN120)
**Meropenem** (MEM-10)	**Azithromycin** (AZM-5)
**Colistin**(CLM-10)	**Nitrofurantoin** (F300)
**Trimethoprim** (TMP5)	**Tetracycline**(TE-30)
**Cefepime**(FEP-30)	**Ampicillin/sulbactam**(A/S20)

Figure 3s

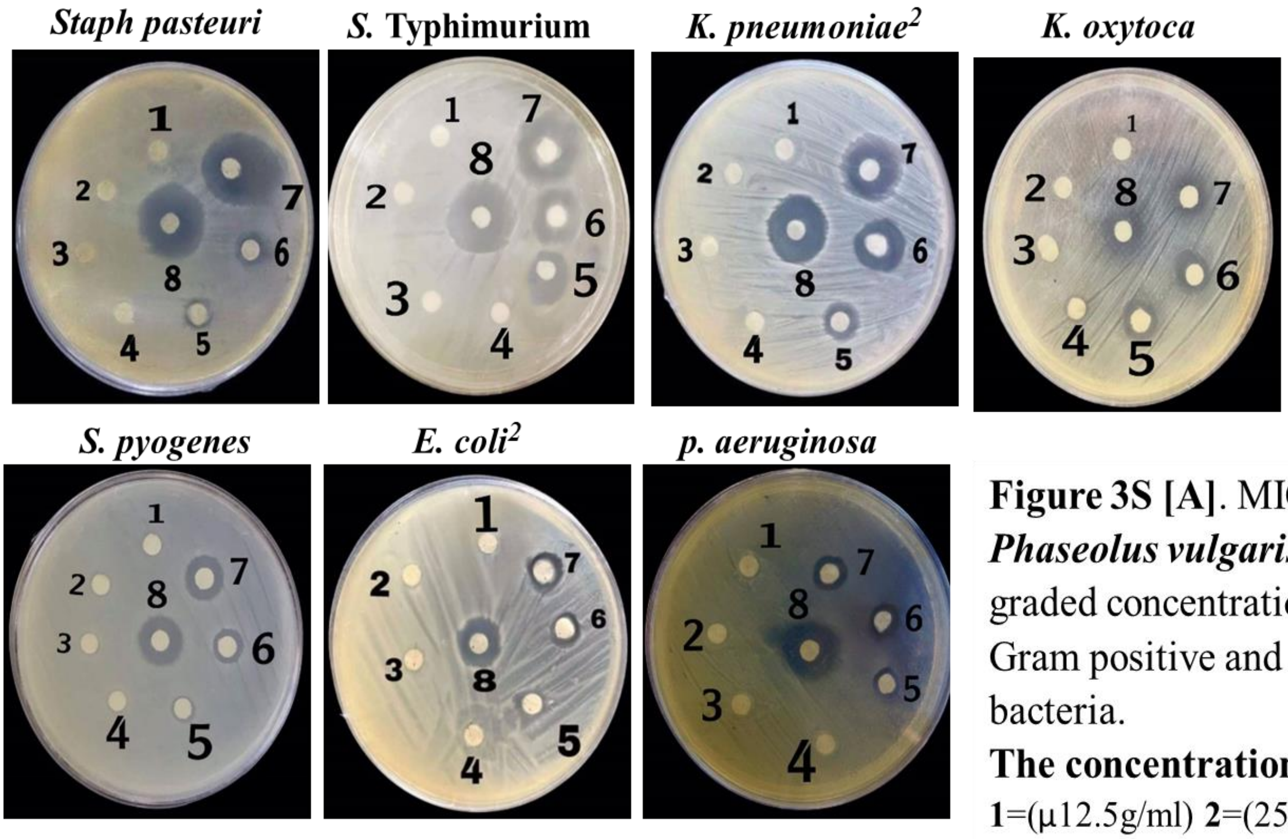

Figure 3S [A]. MIC of **native** ***Phaseolus vulgaris*** seed protein at graded concentration against Gram positive and Gram-negative bacteria.

The concentration codes.

1=(µ12.5g/ml) **2**=(25µg/ml) **3**=(50µg/ml) **4**=(100µg/ml) **5**=(200µg/ml) **6**=(400µg/ml) **7**=(800µg/ml) **8**= (1000 µg/ml)

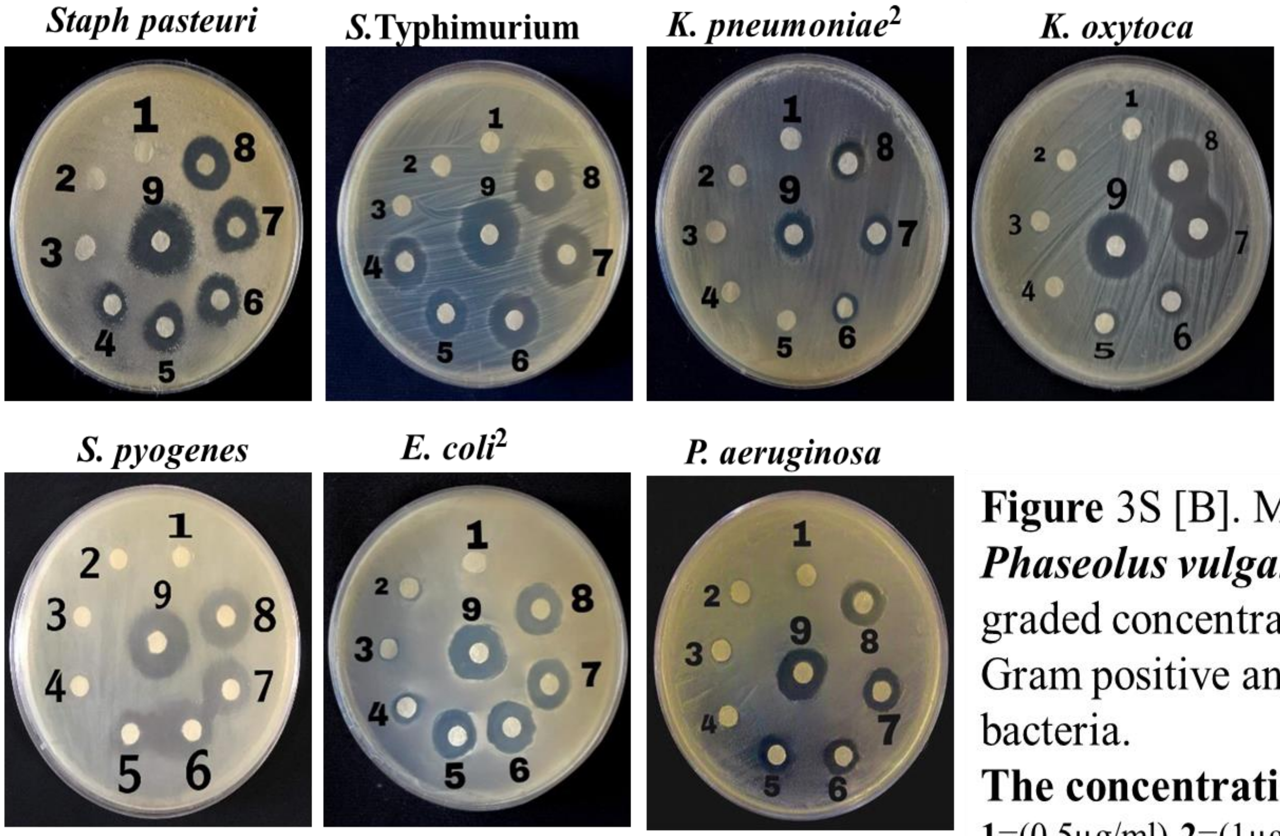

Figure 3S [B]. MIC of **modified** ***Phaseolus vulgaris*** seed protein at graded concentration against Gram positive and Gram-negative bacteria.

The concentration codes.

1=(0.5µg/ml) **2**=(1µg/ml) **3**=(2µg/ml) **4**=(5µg/ml) **5**=(10µg/ml) **6**=(20µg/ml) **7**=(50µg/ml) **8**= (100) µg/ml)

Figure 4s

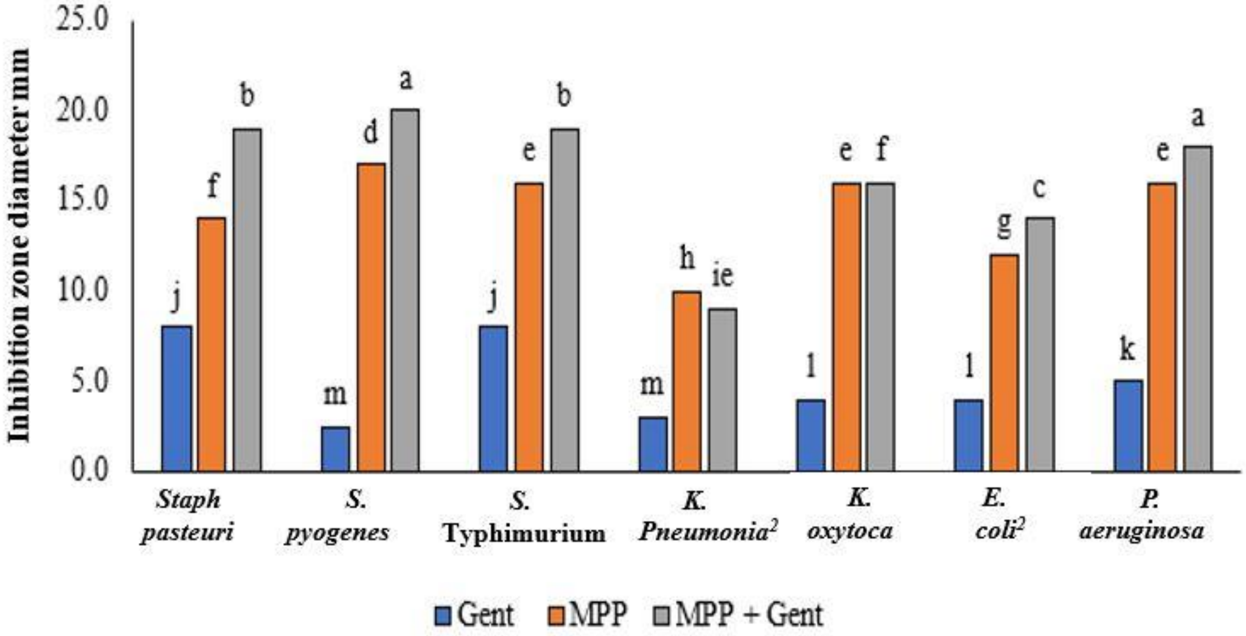

Activate W

Table 1S. Comparison between antimicrobial activity of gentamicin and MPP:

Microorganism	Inhibition zone diameter, mm		Microorganism effect
	Gentamicin disk (10 µg)	MPP (10 µg)	
Staph pasteuri	20.0 d ± 0.25	25.0 a ± 0.06	22.5 a ± 1.12
S. pyogenes	17.0 g ± 0.06	21.0 c ± 0.15	19.0 d ± 0.9
**S. Typhimurium**	8.0 i ± 0.12	22.7 b ± 0.34	15.3 f ± 3.28
K. pneumoniae[2]	0.0 j ± 0	15.0 h ± 0.06	7.5 g ± 3.35
K. oxytoca	19.0 e ± 0.15	20.0 d ± 0.06	19.5 c ± 0.24
E. coli[2]	23.0 b ± 0.26	20.0 d ± 0.1	21.5 b ± 0.68
P. aeruginosa	16.9 g ± 0.12	18.0 f ± 0.12	17.5 e ± 0.24
Antibacterial effect	14.8 b ± 1.66	20.2 a ± 0.67	

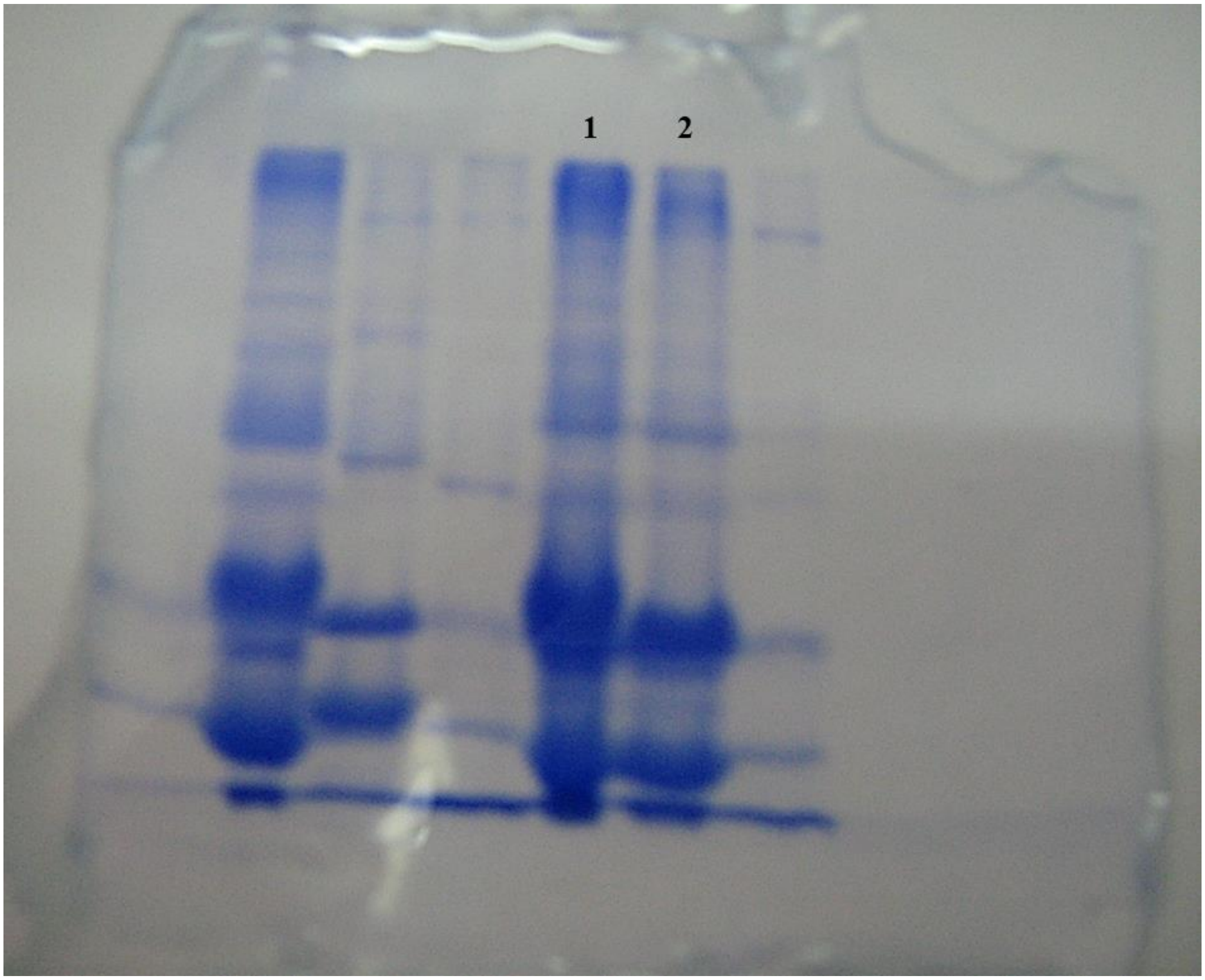

Original SDS-PAGE of Figure 1 (B). The lanes 1 and 2 corresponding to samples 1 and 2 were cropped and integrated in Figure 1 (B)
